# Prominent fin-contributed swimming in squid (*Loligo forbesii*) supports efficient movement in seamount habitats

**DOI:** 10.1098/rsos.250321

**Published:** 2025-07-23

**Authors:** Seth Cones, Nathan Formel, Jorge Fontes, Pedro Afonso, K. Alex Shorter, Gonçalo Graça, C. Robert Priester, Diya Das, T. Aran Mooney

**Affiliations:** ^1^ Department of Biology, Woods Hole Oceanographic Institution, Woods Hole, MA, USA; ^2^ Institute of Marine Sciences—OKEANOS, University of the Azores, Horta, Portugal; ^3^ Institute of Marine Research—IMAR, University of the Azores, Horta, Portugal; ^4^ Department of Mechanical Engineering, University of Michigan, Ann Arbor, MI, USA; ^5^ Flying Sharks, Horta, Portugal

**Keywords:** kinematics, behaviour, respirometry, energetics

## Abstract

Animal movements and the associated energy costs dictate an individual’s scope for activity and habitat use. Yet *in situ* measurements of movement often fail to quantify whole-body movement and their physiological costs. These challenges lead to data gaps connecting how movement behaviours and energy output interact to constrain species’ biogeography. Here we combined swim tunnel respirometry and multi-positional field biologging data to estimate the energy output of squid (*Loligo forbesii*), an ecologically key marine invertebrate. Laboratory respirometry experiments revealed a strong correlation between body mass and metabolic rate during fin-contributed swimming, enabling energy cost estimates in the wild. Free-ranging squid enacted dynamic and diverse fin and jet swimming that varied on short time scales. Animals largely selected (66%) low-amplitude fin-contributed movements where fin waves propagated metachronally. Higher amplitude fin and jet movements were rare, accounting for 4% of time budgets. Application of the bioenergetic model on naturally exhibited behaviours estimated that animals consumed 3117 ± 532 mg O_2_ per day to fuel the predominant metachronal fin movements, an expenditure energetically comparable to that of similar-niche fishes. These unique data reveal substantial behavioural flexibility and indicate squid prefer low-cost movement behaviours that may enable squids’ high growth rates and successful competition with fishes.

## 1. Introduction

How animals move is central to their physiology and ecology. Movements enable individuals to locate suitable habitats, mates and forage, yet these active behaviours incur energy costs that comprise significant portions of animals’ energy budgets [1,2]. Since movement is fundamental in many vital life functions, it necessitates an integrative approach to investigate species bioenergetics, kinematics, behaviour and habitat use [3]. Biologging has emerged as a gateway to remotely track animal movements across these interconnected themes [4,5]. Yet, these four data types (energetics, kinematics, behaviour and environment) are rarely examined holistically for free-ranging animals, and existing data largely focus on higher trophic levels (e.g. cetaceans and sharks). Data on central trophic level taxa, and invertebrates in general, are lacking [6]. Historically, these data are difficult to collect for soft-bodied invertebrates, such as keystone squid, yet are particularly vital since they comprise a high biomass across the oceans [7], support substantial global fisheries [8], and their behavioural and physiological plasticity to environmental change can have ecosystem-level impacts [9–11]. Given their vital role and the stresses facing the marine environment, there is a clear need to quantify squid swimming behaviour and specific energetic output of these keystone animals under naturally varying environmental conditions *in situ* to better predict population responses in changing oceans.

Squid, and cephalopods broadly, are undergoing a multidecadal increase in abundance and are often considered ‘winners’ as the climate and oceans change [12]. They are diverse and successfully compete with marine fishes in habitats from intertidal pools to the deep abyssopelagic [13]. The underlying mechanisms for their success within varying biomes are multifaceted and likely involve their live-fast die-young life history, high fecundity and high behavioural and phenotypic plasticity [12,14]. Traditionally, squids’ success has been surprising considering their putative physiological inefficiencies and propulsive constraints relative to marine fishes [15]. Squid are thought to expend more energy to locomote, only to perform worse compared to pelagic fishes in the same niche [2,16–18]; but see [19]. For example, laboratory data show that despite having a higher cardiac output, heart rate, oxygen consumption and oxygen extraction from the blood, loliginid squid have lower critical and sustainable swimming speeds than fishes of similar lifestyles [20]. Although loliginid squid may not be suited for life in the vertebrate fast lane, recent laboratory data have revealed that propulsive and muscle contractile efficiencies are similar or better than vertebrate muscles [21]. This suggests that cephalopods’ high swimming costs stem from high-power mechanical requirements [21–23]. These energetic observations raise questions of how individuals are moving *in situ*, and what behavioural mechanisms may be compensating for their other physiological disadvantages and high mechanical power requirements. In essence, what movement patterns support their potential success?

As some of the first ‘swimmers’ to rise off the seafloor, cephalopods relied on buoyancy and primitive jet propulsion (shell pumping) to navigate the benthopelagic environment [24]. While no extant cephalopod has abandoned the jet propulsion system, squids evolved another propulsor: extremely diverse muscular fins [25]. Having two spatially isolated propulsors grants exceptional behavioural capacities. Squid, unlike most fishes, can swim bidirectionally and directed powered jets that allow them to rapidly accelerate nearly omnidirectionally [26]. Jet propulsion involves contractions of the circular mantle musculature to expel water through a funnel. These mantle contractions support movement through the water, yet these mantle contractions and expansions are constant and enable gill ventilation [27,28]. Squid fins are employed in concert with rhythmic jets. Fin-contributed locomotion is enabled by either fin flapping movements or metachronal fin waves that propagate bidirectionally along the mantle [25,29]. Metachronal fin-contributed swimming is more continuous and economical compared to jet propulsion alone, and it is integral to maintain thrust and efficiency while squid are refilling the mantle for jet propulsion [29,30]. Laboratory studies of the brief squid (*Loliguncula brevis*) in swim tunnels have demonstrated that fins can contribute the majority of thrust (up to 83%) across a wide range of swimming speeds [22]. Despite this significant contribution, energetic studies of free-ranging animals have been limited and only sought to quantify and describe jet propulsion behaviours [2,25,31], and there are no data on fin movements of free-ranging animals. This leaves open fundamental, yet key questions such as (i) what are the bioenergetic costs of fin-contributed movements, (ii) how often do the animals employ fin-contributed locomotion and (iii) do animals use a range of fin movement strategies in the wild?

Observing movement patterns and gaits of free-ranging squid is challenging. Epi- and mesopelagic loliginid squids inhabit high-pressure and low-light habitats, and they are highly mobile making direct observations unfeasible. The development of sensing technology such as the ITAG (Invertebrate Tag [32]) and BIMS (Bioadhesive Interface for Marine Sensors [33]) enables collection of fine-scale movement data using high-frequency sensors that do not affect the movement of these fragile, soft-bodied taxa [34]. These and similar technologies have provided novel data on loliginid and ommastrephid squids, revealing dynamic postures, swimming speeds and jet propulsion behaviours [35–37]. These biologging tags measure movement at the point where the tags are affixed on the body. However, understanding how fin movements and jet propulsion contribute to overall locomotion requires direct measurements of multiple body positions. The inability to measure two body positions with a single sensor results in a limited view of the whole-body kinematics and associated energetics. As such, new measurement types are needed to quantify coordinated kinematics of free-ranging animals.

Here, we investigated the behaviour, kinematics and the associated energetic cost of free-ranging veined squid (*Loligo forbesii*) in waters off the Azores archipelago, Portugal. Veined squid are a benthopelagic coastal species and are a key protein source for many local and migratory megafauna [38–40]. Squid are known to aggregate within the shelf between Faial and Pico Islands where water currents can exceed 1.0 m s^−1^, and the uneven bathymetry produces large internal waves [25,41]. Although *L. forbesii* are restricted to coastal shelf slopes, they likely actively swim to contend with and navigate strong physical currents to maintain position within suitable habitats. To collect concurrent kinematic, behavioural and physiological information, we integrated biologging, magnetometry and respirometry data to measure precise fin movements in the field and mapped the observed movement to energetic costs using metabolic experiments with confined animals. By combining these approaches, we were able to provide the first insights into jet and fin kinematics of a free-ranging, ecologically key cephalopod, showing that low-intensity metachronal fin-contributed swimming is economical and dominant across daily time scales.

## 2. Methods

### 2.1. Animal collection and tagging

All animals used in this study were caught on the northern and southeastern coast of Faial Island, Azores archipelago (38.3003° N, 28.3522° W) in May 2023. Care was taken during capture and handling of animals to limit physical stress. Animals were caught via jigging at roughly 250 m water depth and were slowly brought to the surface with an electric fishing reel. Jigging is a minimally invasive means of capture since it reduces damage to the fragile epidermal layer [42]. Once at the surface, animals were hoisted into the boat with a soft silicone net. Squid collected for laboratory respirometry experiments were transferred to the Porto Pim Aquarium in large, aerated coolers. Squid used for field tagging were directly transferred to a padded table equipped with constant ambient seawater flow to ventilate the gills. Squid eyes were covered to reduce light stress during the tagging process [43].

Wild (*n* = 5) and laboratory (*n* = 7) squid were equipped with a single ITAG, which is a modular archival biologging tag designed for marine invertebrates [32]. It contains a high-frequency (100 Hz) accelerometer, magnetometer and gyroscope as well as a lower-frequency (1 Hz) light, temperature and pressure sensor. ITAGs were affixed 5−10 cm from the posterior mantle edge using either BIMS, which is a rapid and flexible adhesive for soft marine invertebrates [33], or dissolvable surgical sutures. The ITAG form used in the present study was designed specifically for squid; its interfacial edge is contoured to match mantle curvature, and its hydrodynamic shape limits additional lift and drag forces to the animal. ITAGs released from free-swimming squid by triggering an electrical current onto a nichrome wire that held the sensor and base plate components together. After the wire corroded, the fractionally buoyant sensor floated towards the surface where it was recovered via an emitted radio signal (Advanced Telemetry Systems). Animals tagged in captivity resumed normal swimming less than one minute after tagging and conspecifics showed no antagonistic behaviours towards the focal animal [32,34]. Additionally, one wild tagged *L. forbesii* in previous field expeditions was caught by a handline jigging fisherman within 2 hours of release, demonstrating that animals have rapidly recovered and resumed active foraging shortly after field tagging.

### 2.2. Fin magnetometry

**Figure 1.**
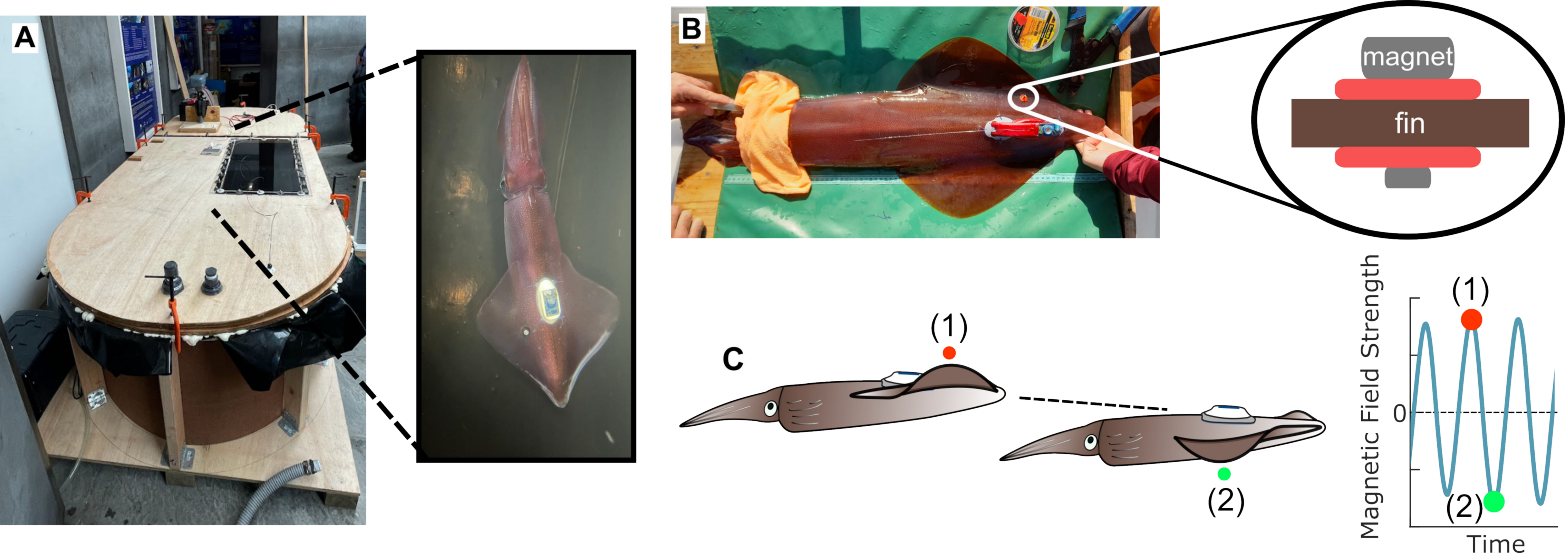
Peripheral fin movements can be detected using magnetometer magnet coupling. (A) An image of the custom-built swim tunnel respirometer chamber and an experimental animal affixed with an ITAG and fin magnet swimming within the chamber. (B) A photograph of one field animal equipped with ITAG and fin magnet before immediate release back into the sea. A magnet was affixed to the squid fin by gluing it to a stainless-steel wire that passed through the fin musculature and crimped on ventral surface. The magnet and crimp were secured with silicone washers to ensure only soft surfaces were in contact with the skin layer. (C) A schematic illustrating how the tight coupling of the fin and magnet interacts with the ITAG magnetometer to measure fin movements. During the fin upstroke, the magnet (red) is positioned more dorsally and closer to the ITAG magnetometer, thus producing a greater magnitude magnetic field strength. Conversely, during the fin downstroke, the magnet is farther from the magnetometer, which produces a relatively lower magnitude magnetic field strength. The continuous transition between these positions results in a sinusoidal curve in the magnetic field strength data whose frequency is equivalent to the finning rate.

To measure squid fin movements, we affixed a small cylindrical magnet on one lateral fin (figure 1; magnet 2 × 10 mm^2^, 2.00 g). Magnets were attached to a stainless-steel wire inserted through the fin musculature near the fin–mantle connection to limit torque on the fin. Soft silicone washers were placed on either side of the fin to limit any physical stress, and the wire was crimped to prevent any shifting of the components (total weight: 5.62 g). As a result, the magnetic field strength was tightly coupled to the fin position, and movements by the fin distorted the magnetic field at a frequency equivalent to the fin rate. For example, during the fin upstroke, the magnet was positioned more dorsally and relatively close to the ITAG magnetometer, which produced a higher magnitude magnetic field strength value. Conversely, during the fin downstroke the magnet was more ventral away from the ITAG resulting in lower magnitude magnetic field strength values (figure 1C). Continuous metachronal fin waves produced sinusoidal signals in the magnetometer data, which could be mapped back to fin position [44]. However, since fin position and magnetic field strength relationships were not calibrated, specific fin angle measurements were not possible and only ‘relative’ fin positions were reported.

The tight coupling of the fin and magnet together with the high sampling rate of ITAG facilitated an automated approach for the identification of fin movement signals. Individual finning events (i.e. one fin upstroke and downstroke) were enumerated through a trained peak detecting algorithm (MATLAB findpeaks.m). Absolute values of the three magnetometer axes (*x*, *y* and *z*) were summed, and a moving average filter (window size: 30) was used to smooth the signal. Next, a validation and peak detection optimization framework was established using manually audited finning signal data. Magnetometry data were visually scanned to find three time periods in which squid exhibited diverse fin behaviours to account for fin signals under multiple behavioural states and limit any bias. For each segment (3 minutes), the number of peaks likely resulting from fin activity was manually counted and served as training data to optimize parameters of the findpeaks.m function (minimum peak prominence, minimum peak width and maximum peak width; see electronic supplementary material, S1). For each 3-minute segment, custom MATLAB code looped through 60 372 different parameter settings and indexed parameter combinations that successfully marked 100% of the manually annotated fin crests. After, all successful parameter settings from the three segments were averaged, and the peak detector was applied to the entire deployment.

Magnetometer signal strength varied slightly as an animal changed its orientation. Minor post-processing was needed to filter out signal peaks that resulted from orientation changes instead of finning events. Detected finning events (i.e. magnetic field strength peaks) with an inter-fin interval, or time between subsequent fins, of greater than 4 seconds both before and after a fin crest were removed. These isolated crests in the signal were likely due to lower-frequency orientation changes rather than one higher-frequency fin upstroke and downstroke. Yet, these artefacts were important to remove since a detected magnetic field signal peaks from an orientation change would shift a behavioural classification away from gliding to an active state (more below).

### 2.3. Respirometry

To measure metabolic rates during fin-contributed swimming, individual squid were isolated in a large (1485 l), custom-built swim tunnel respirometer system. The system design was similar to the widely used Steffensen-type swim tunnels (electronic supplementary material). These are oval track systems with separate areas for water current generation, an animal working section and two curved flow channels with mesh to encourage laminar flow. The base of the system was constructed out of plywood which supported a pool liner to contain seawater. Polypropylene sheets served to separate the motor propellor from the working section and additional sheets were curved to support laminar flow between these two volumes. An electric kayak motor (Newport Kayaks) was integrated through the system lid to generate current. The kayak motor had preset levels (1–5), which allowed discrete and comparable treatments across animals. For each motor level, the flow speed was calibrated at five different points along the central chamber cross section using an impellor flowmeter (Flinn Scientific). Flow speeds increased (0.16 ± 0.04, 0.18 ± 0.04, 0.24 ± 0.05, 0.27 ± 0.06, 0.38 ± 0.1 m s^−1^) from motor levels 1 to 5. One oxygen sensor (PyroScience OXROBSC) was positioned upstream and one downstream from the working section with a temperature gauge (PyroScience TDIP15) to measure oxygen uptake at 1 Hz throughout the experiments.

Prior to experiments, animals were held in a large display tank (8 × 4 × 2 m^3^) and fasted for at least 24 hours. Natural seawater from the adjacent Porto Pim Bay (near the capture location) was supplied to the holding and experimental tanks. Each experiment day, one squid was removed from the holding tank and quickly tagged with an ITAG via suturing or the BIMS. The squid was then transferred into the working section of the respirometer, and the observation window was covered with a towel to allow animals to acclimate for 30 minutes in low light levels. The motor was set to level 1 (0.16 m s^−1^) to provide gentle current for squid to orient within. Squid often swam erratically when water flow within the animal chamber was stagnant. After the recovery period, a random motor level was selected and maintained for 30 minutes. The observation window remained partially or totally covered throughout. Next, the motor was reset to level 1 for a 15 minutes rest period. No squid became exhausted and were all able to maintain position within the working section, so post-exercise oxygen consumption was not considered during the 15-minute rest periods. This process was repeated at random motor levels, resulting in up to three trials (i.e. speed exposures) per animal. Systems were partially flushed with seawater during rest intervals to prevent oxygen levels from decreasing below 75% air saturation [45]. Experiments were conducted at only one narrow temperature range (18.8 ± 0.46°C). After each experiment, wet weight was measured and the tag was removed before experimental squid were released into waters adjacent to the aquarium. Experiments were conducted at surface pressure since hydrostatic pressure does not strongly influence squid metabolic rate [46].

Additionally, measurements of the microbial respiration (i.e. decrease in oxygen without animal present) of the water were taken at the beginning of each experiment day. These oxygen consumption rates were subtracted from animal measurements to determine the oxygen uptake of the animal during swimming. Metabolic rate (mg O_2_ h^−1^) was calculated by *M* = ∆VO_2_/time using regression analysis of each treatment data series. Then, to normalize to mass-specific metabolic rates (mg O_2_ kg^−1^ h^−1^), we divided *M* by the squid wet mass. Dissolved oxygen traces during both experiments and controls were linear (*r*^2^ ≥ 0.98) demonstrating the system was completely sealed with no oxygen leakage or intrusion.

### 2.4. Gait identification and classification

Squid use both fin and jet propulsion for locomotion, and fin movements can be enacted in synergy with the jet propulsion system. This fin-contributed swimming was identified and quantified through magnetometry (see §2.2). Jet propulsion via mantle contraction and expansion is inherently pulsatile, and jetting behaviours were inferred from both accelerometry and magnetometry. Previous data show high-amplitude jet propulsion events from induced escape responses can be classified using dynamic acceleration thresholds [34]. These jets differ from low-amplitude jetting continually employed during slow swimming and respiration.

To parse fin-contributed and high-amplitude jet behaviours from the ITAG’s inertial motion sensor outputs, data were initially segmented into 1 second windows of concurrent fin position and dynamic surge acceleration, or acceleration due to animal movements along a squid’s long axis (see [34] for full description of accelerometry filtering). For each 1 second window, data were scanned for at least one finning event or surge acceleration levels exceeding a 0.10 g threshold [35]. If either condition was met, the animal behavioural state was labelled ‘active’ since either the fins were active or there was a high-amplitude jet. ‘Inactive’ states were periods of no fin movements and surge acceleration levels less than 0.10 g for at least 5 consecutive seconds. Low acceleration and lack of fin movements suggests that animals were largely inactive, although the jet propulsion system remained active. Inactive states were classified as ‘gliding’ since there was no evidence of squid resting on the substrate.

Active swimming behaviours with detected fin movements and/or high-amplitude jets were classified into movement behaviours by their dominant frequency components determined by fast Fourier transforms (FFTs). First, active fin position data were compiled into continuous 5-second windows. Next, a Hanning window was applied to limit spectral leakage, and the windowed data were zero padded with 10 000 zeros to increase the output frequency resolution. An FFT was then applied to identify the dominant movement frequency for each window. Frequency distributions were consistently bimodal across animals; the local minimum was used to distinguish between a higher-frequency metachronal fin-contributed swimming behaviour and lower-frequency movements. Low-frequency movements containing surge acceleration peaks greater than 0.1 g were classified as ‘jets’, and segments with accelerations below 0.1 g were classified as ‘other’. ‘Other’ swimming behaviours were lower-frequency fin movements where the jet system did not produce body accelerations typical of high swimming speed movements measured in tanks and the field [34,35]. However, some high-frequency fin sequences also included brief, high-acceleration jet surges, highlighting that high-amplitude jets can contribute to propulsion even during metachronal fin movement.

### 2.5. Bioenergetics of animals *in situ*

A log–log linear model was constructed to describe the relationship between wet body mass and mass-specific routine metabolic rate [47]. This laboratory-calibrated metabolic model was used with *in situ* biologging data to estimate energetic cost of similar active behavioural states. Less active gliding behaviours were not elicited in the respirometer, and thus we could not incorporate the behaviour into our model. Instead, we averaged previously calculated standard metabolic rate (86 mg O_2_ kg^–1^ h^–1^) and metabolic rate while hovering (183 mg O_2_ kg^–1^ h^−1^) as a proxy for the energetic cost of gliding [2,25]. Both finning and gliding metabolic rate measurements estimate only the cost of movement and basal metabolism and assume the animals are not repaying an oxygen debt or spending energy to digest food. Additionally, incorporating standard metabolic rates for gliding costs assumes no postural costs, which can be non-negligible for negatively buoyant animals [19,48].

Squid are ectothermic and their metabolic rates are tightly linked to ambient temperature. To account for metabolic rate changes with temperature (field: 14.5 ± 0.68°C; laboratory: 18.8 ± 0.46°C), a q10 coefficient of 2 was applied [16,47]. This coefficient describes the magnitude of metabolic rate change across a 10°C range. In this context, the estimated metabolic rate of a free-ranging squid was adjusted using the following equation:


$$M_{corr}=M\times q10[(T2-T1)/10],$$

where *M*_corr_ is the metabolic rate correction for temperature, *M* is the metabolic rate estimated from metabolic model, *T*1 is the temperature during respirometry experiments, and *T*2 is the ambient temperature at the squid depth.

## 3. Results

### 3.1. Respirometry

**Figure 2.**
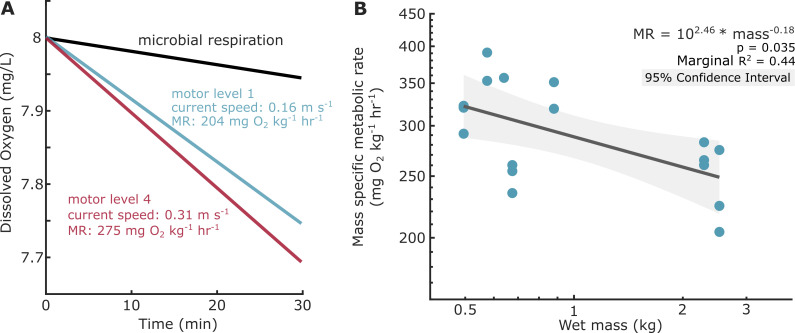
Mass-specific routine metabolic rate scales with squid body mass. (A) Linear models constructed from dissolved oxygen curves during control microbial respiration (black) and two swimming trials (blue, red) at different flow speeds. (B) Mass-specific routine metabolic rate (mg O_2_ kg^−1^ h^−1^) was negatively correlated with body mass (both the *x* and *y* axes are log scale).

Seven respirometry experiments were conducted (dorsal mantle length (DML): 26−54 cm; (mean ± s.d.) mass: 1.15 ± 0.86 kg), encompassing a broader size range of squid tagged in the field. The number of speed exposures for each animal varied from one to three, for a total of 17 swim trials at varied flow speeds. Squid within the respirometer solely enacted a fin-contributed swimming mode where the fin waves propagated metachronally along the mantle cavity to propel in either the arms first or mantle first direction. Data from both swimming orientations were combined. There were no glides or high-amplitude jet propulsion behaviours where fins remained wrapped around mantle cavity. The gradient of current speeds induced varied swimming intensities measured as squid routine metabolic rate during low and intermediate speeds, although exact swimming speed was not measurable due to minor differences in flow speed and low light levels within the covered test section. Squid wet mass was negatively correlated with mass-specific routine metabolic rate in the range of 0.495 to 2.525 kg (log–log linear mixed effects model, d.f. = 15, marginal *r*^2^ = 0.44, *p* = 0.035; figure 2). Within this range, mass-specific routine metabolic rate ranged from 204 to 391 (mg O_2_ kg^−1^ h^−1^).

### 3.2. *In situ* behaviour across time scales

**Figure 3.**
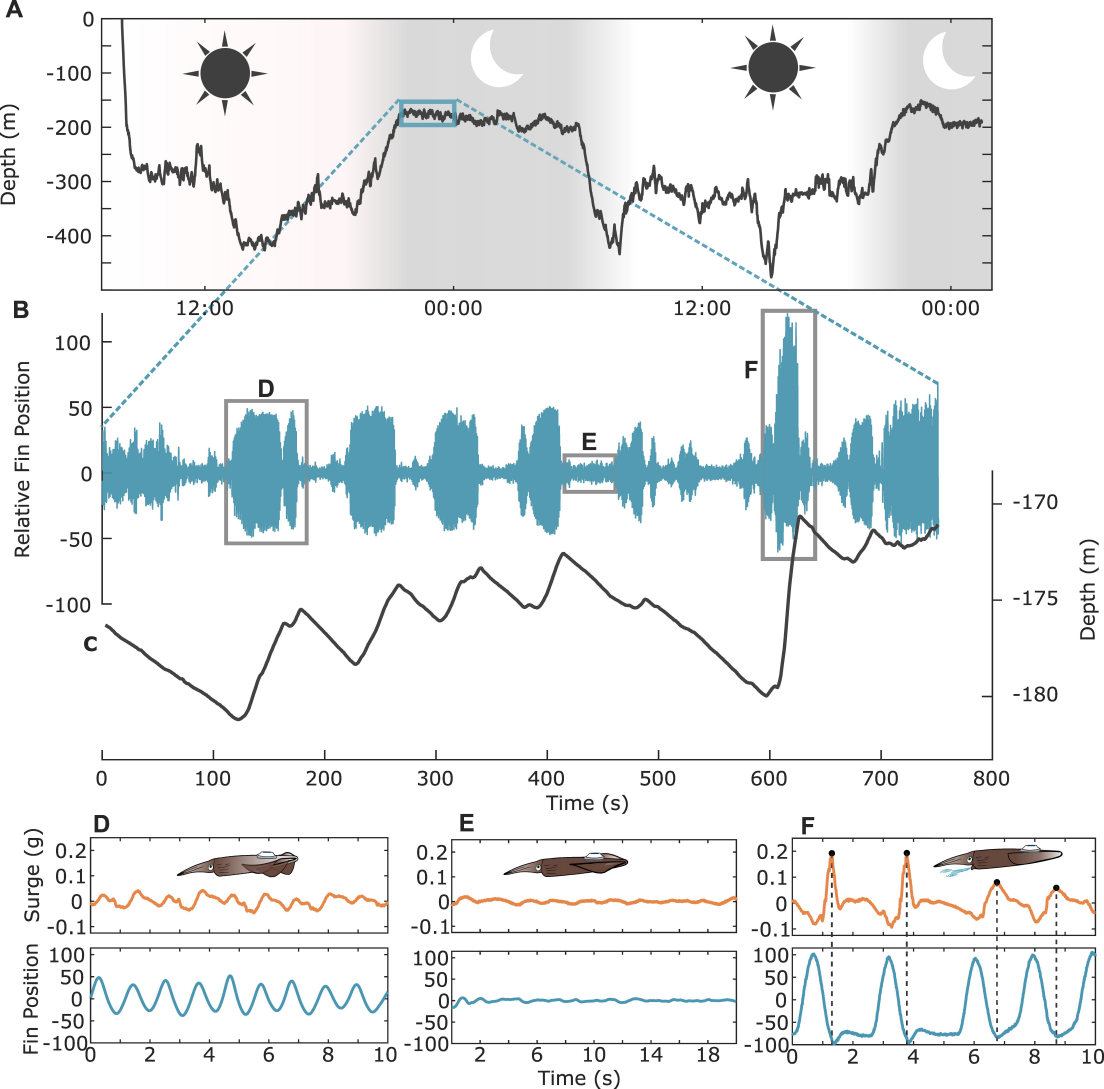
Squid fin behaviours were diverse and facilitate dynamic movements in nature. (A) A dive profile of one animal, which occupied different vertical layers during day and night periods. (B,C) Concurrent fin position (blue) and depth (black) data shortly after upwards vertical migration. The animal was climb-and-glide swimming, and different phases were characterized by finning signals of differing amplitudes. (D) Surge acceleration (orange) and fin position data (blue) during a metachronal finning sequence where fin position continuously moved dorsoventrally. (E) A second example gait where surge acceleration and fin position remained steady for 20 seconds. It is likely the fins remained extended in the lateral plane to generate lift and reduce sinking rate. (F) A third gait example where high-amplitude fin behaviours resulted in high-amplitude forward propulsion. The peak acceleration coincided with the lowest fin position indicating the jet propulsion system was enacted in coordination, and its relative contribution can vary as shown by varying surge peaks in the later portion of the sequence.

In total, we collected 164 hours of high-resolution *in situ* movement data from five animals (mean = 33 ± 24 hours per animal) spanning a wide size range of squid sizes (DML: 34−52 cm; mass: 1.14 ± 0.52 kg). Tag attachments were conducted quickly, taking no longer than 6 minutes. After release, animals rapidly descended to roughly the same depth as capture (typically around 250 m). Animals generally occupied shallower vertical layers during night periods (figure 3A), and migratory times closely aligned with sunset and sunrise.

Movements of the fin and fin magnet produced clear signals in the magnetometer data (figures 1 and 3). Fin amplitudes were of noticeably different magnitudes across different swimming behaviours, varying across scales of seconds (figure 3B,C). Cyclic climb-and-glide swimming was prevalent throughout all portions of the day. Fin movements were higher in amplitude during climbing periods, compared to gliding periods, which were often, but not always, inactive periods with no fin movement (figure 3B,E).

Concurrent acceleration and fin position uniquely revealed a diversity of *in situ* swimming modes. First, continuous ‘finning’ times were periods when squid fin waves propagated metachronally (figure 3D). Second, ‘gliding’ was inactive behaviours where both acceleration and fin position were near zero for extended durations (figure 3E). Low acceleration and constant fin position indicated animals were not generating fin-mediated propulsive forces for locomotion and that the fins were likely extended laterally to enable soaring. Intense ‘jetting’ movements were also present, and these were often, but not always, accompanied by a high-amplitude fin upstroke and downstroke (figure 3F). Instead of being continuous signals like metachronal finning, the squid fins remained low and were likely wrapped around the mantle for 1−1.5 seconds before the next sequence. Fin manoeuvres were coordinated with the jet propulsion system. Acceleration peaks were prominent during these jets, and, notably, the acceleration signal consistently peaked at the end of the fin downstroke, which indicates strong coordination between the jet and fin propulsors. The magnitude of the jet contribution varied with some body accelerations surpassing 0.2 g, while others were near 0.1 g despite similar fin movements (figure 3F).

### 3.3. Gait identification and classification

Our peak detection algorithm found and parametrized a total of 419 412 individual fin events (electronic supplementary material, figure S1). We found 9247 inactive gliding sequences (no fin usage and low acceleration) constituting 18% of time budgets. No inactive periods were of constant depth, suggesting animals were not bottom sitting. Instead, during inactivity, squid showed negative depth changes indicating apparent gliding downwards in the water column (figure 3C). Inactive gliding sequences tended to be brief (13.2 ± 10.7 seconds) but could be as long as 157 seconds.

**Figure 4.**
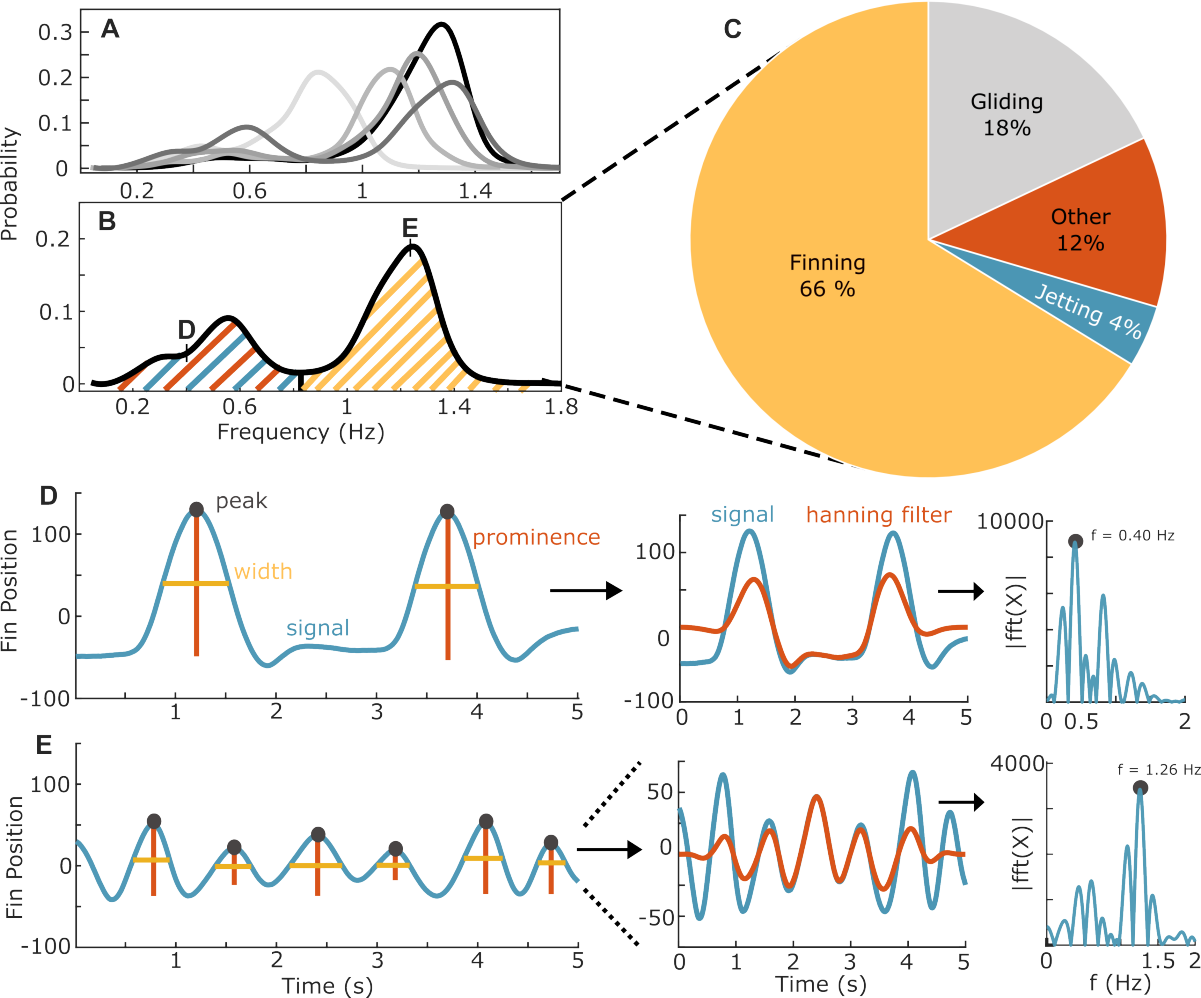
Metachronal finning dominates squid time budgets. (A) Gait frequency probability distributions during active behavioural states overlaid for each animal, and (B) one example distribution to illustrate how gaits were distinguished by frequency. Each distribution is bimodal with a prominent peak in the higher frequencies representing finning activity (yellow) and lower-frequency peaks corresponding to jet dominated (blue) and other sequences (orange). (C) Cumulative time budgets of all experimental animals. (D,E) An expanded view of how signals were characterized. Active data were sequenced into 5 second windows and transformed with a Hanning filter (orange) and zero padding (not shown). Next, a fast Fourier transform was run on the filtered sequence, and the peak frequency was extracted and used to characterize the signal.

Animals were active 82% of the time, and these 5-second sequences were further divided into swimming modes by their frequency and amplitude (electronic supplementary material, figures S1 and S4). Analysis of all active data sequences revealed that high-intensity jets comprised 4.2 ± 1.5% of animals’ time budgets (figure 4). Metachronal fin-contributed swimming was substantially more prevalent (66.2 ± 9.4%) and was the dominant mode in every animal’s time budget (figure 4C). A subset of 7.5 ± 4.1% of metachronal fin-contributed sequences also contained high-amplitude surges exceeding 0.1 g, suggesting that high-intensity jets were enacted during both low- and high-frequency fin movements. Finning frequency for all animals averaged 1.12 ± 0.19 fins s^−1^. Mean finning frequency varied with DML, with the largest animal having a lower finning frequency (0.81 fins s^−1^) than the smallest animal (1.27 fins s^−1^). ‘Other’ lower-frequency fin and body movements (<0.1 g) and were elicited 11.6 ± 6.6% of the time.

### 3.4. Bioenergetics of *in situ* movements

**Figure 5.**
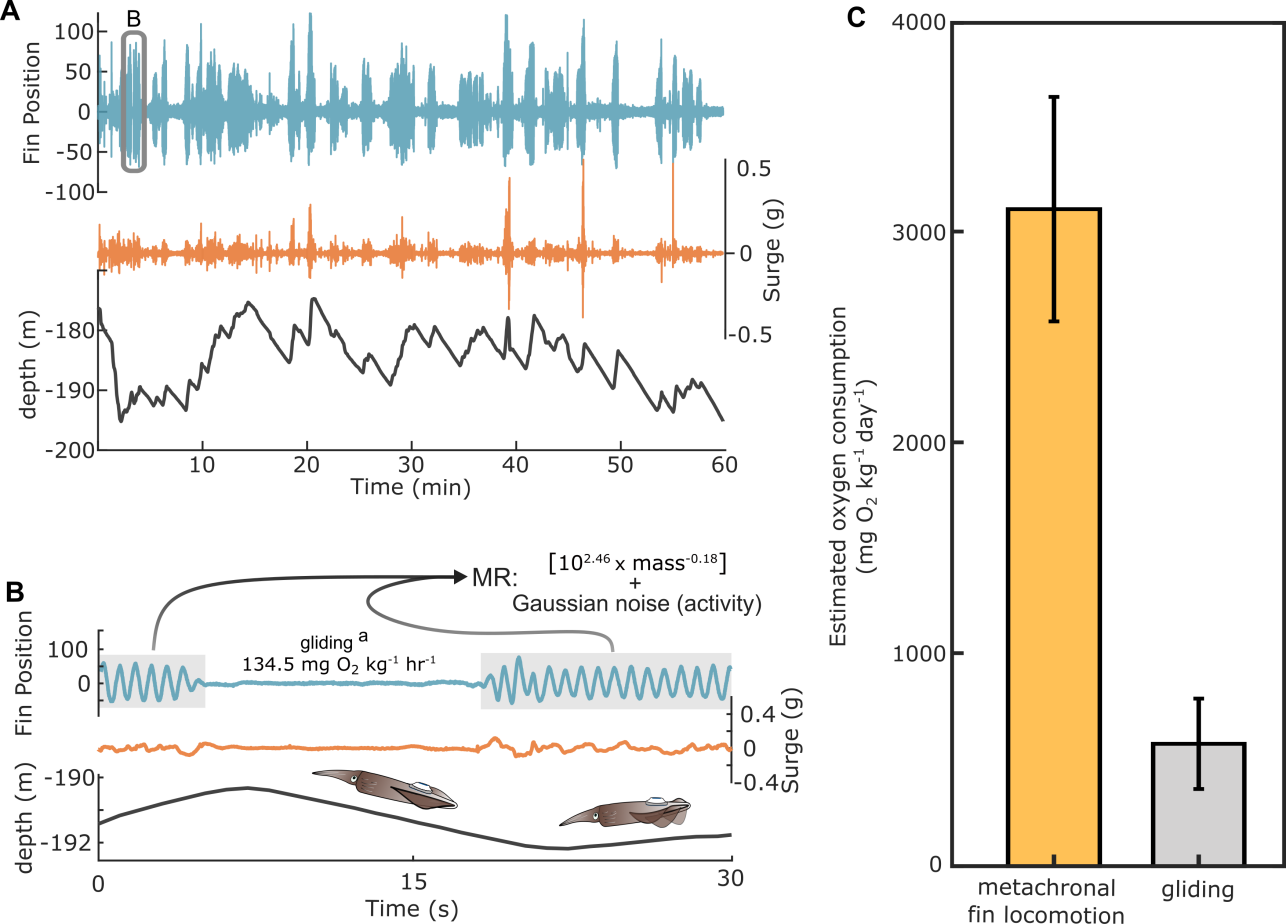
Abundant fin-contributed locomotion and gliding enabled efficient movements *in situ*. (A) Concurrent fin position, acceleration and depth during a 1-hour segment, which are key parameters in behavioural state identification and energetic modelling. (B) An expanded climb-and-glide movement sequence depicting fin positions, surge acceleration, and depth to illustrate how the bioenergetic model was applied to natural movements. (C) Mean and s.d. of metachronal finning and gliding metabolic costs (mg O_2_ kg^−1^ d^−1^) per 24-hour period. ^a^[2,25].

Time sequences identified as low acceleration metachronal fin-contributed swimming and gliding were incorporated into our developed metabolic model to estimate energy cost of free-ranging squid across a 24-hour period (figure 5A,B). Squid consumed an estimated 3117 ± 532 mg O_2_ per day to fuel observed metachronal fin-contributed locomotion, and an estimated 580 ± 214 mg O_2_ per day to glide (figure 5C).

## 4. Discussion

Fins contribute substantially to the overall swimming behaviour of squids, but fin behaviours and the associated energetics of free-ranging animals are not well understood. Here we addressed this knowledge gap using a novel biologging approach to quantify fin-contributed swimming *in situ*, and a data-driven model that maps morphology and fin movements to yield time–energy budgets for a keystone taxon. This investigation has led to three key results. First, a single biologging sensor can be used to characterize and quantify both the movements of the body and a spatially isolated propulsor (i.e. fin). Second, the time budget estimated from the free-ranging *L. forbesii* fin movements indicates that the animals use a range of movement strategies in the wild. Squid fins remain active 82% of the time, and yet low-intensity metachronal fin movements were highly prevalent suggesting animals often strive for low-cost movements. Third, coupled respirometry and biologging revealed that while squid swimming behaviours were extremely diverse in the wild, prevalent fin-contributed swimming resulted in metabolic costs comparable to those of many marine fishes.

### 4.1. Time budgets

Despite the fins playing an integral role in the overall propulsion of squid in laboratory studies [22,29,49], there were no quantitative data on fin movements of free-ranging cephalopods. Instead, often broad characterizations of estimated fin usage have been hypothesized based on a species morphology and habitat use [25]. Here, we showed, using multi-positional magnetometry-biologging measurements, that low-amplitude metachronal fin-contributed swimming dominated *L. forbesii* time budgets *in situ* (66%) supporting the importance of this behaviour to squid mobility and movement. Predominance of movement patterns that generate low-amplitude accelerations is consistent with O’Dor *et al*. [50], who used active tracking and intra-mantle pressure sensors to measure the frequency and amplitude of jets of *L. forbesii* in the Azores. Similarly, they found a majority (70%) of mantle water pressures were consistent with low-speed swimming and hovering, and vastly different from escape jets and dynamic acceleration events [34]. Fins are thought to be heavily involved in low-speed swimming and manoeuvring, and they also increase stability [51]. This *L. forbesii* population is benthopelagic, often swimming within a few metres of the substrate [25,50]. Considering the high-energy hydrodynamics around the Azores archipelago, *L. forbesii* may use their fins for extensive periods to navigate the energetic tidal currents. Additionally, the rocky volcanic substrata would likely damage their fragile epidermal layer, further supporting prominent fin-contributed swimming to navigate complex habitats [25]. *Loligo forbesii* also use fin movements during diel vertical migrations, depth maintenance swimming and descent swimming suggesting fins contribute substantially in diverse behavioural contexts [35].

O’Dor *et al*. [50] postulated this population soars within tidally generated internal waves. We found no evidence of bottom-sitting behaviours common of other loliginids, suggesting that consistent swimming or gliding was necessary throughout to maintain vertical position in the water column for these negatively buoyant animals. Here, we showed inactive gliding comprised 18% of the animal’s time budgets, and during these periods, fin positions were held steady. Although it is difficult to confirm, it is likely the fins remained extended in the lateral plane to generate lift and reduce the sinking rate. Consequently, these squid may seek out areas of upwelling to dynamically soar similar to birds gliding in updrafts [50]. Because animals did not bottom-sit, this suggests the significant majority of resting periods were during the gliding phase of prominent intermittent climb-and-glide swimming. Gliding periods varied in duration, but in some instances the fins remained active (figure 3B), so there are likely multiple behavioural modes encompassed within gliding movements. Additional data are needed to differentiate subtle behavioural differences.

No cephalopod has abandoned jet propulsion, which may be an evolutionary constraint or the coordination of the fin–jet system is more advantageous than fin propulsion alone [24]. We demonstrated fins are often employed in coordination with the jet during high-intensity movements, where the fin downstroke is likely enacted just before mantle contraction (figures 3F and 4). The contribution of the jet system to overall surge acceleration varied, indicating a high degree of flexibility between fin and jet interactions (figure 3F). Additionally, the graded contribution during jet propulsion suggests the non-giant axon system is employed, at least in part, in conjunction with the distinct fin neuromuscular system [52]. There have been extensive laboratory studies to describe the animal–fluid interactions during fin and jet swimming [26,29,53]. These studies visualize the wake structures shed from the fins and funnel aperture, and while noting high wake complexity, fin and jet wake interactions increase at higher speeds as the angle between the mantle and funnel decreases [22]. As swimming speed decreases, coordination is less apparent. This result was similar to our accelerometry data, where fin and jet coordination was more apparent during high-amplitude swimming modes.

### 4.2. Swimming energetics

Measurements of oxygen consumption during obligatory exercise are difficult to attain for large and fragile marine species and require expensive laboratory equipment. Often, this limits our ability to translate tag-measurable parameters to more ecologically relevant metrics such as oxygen consumption. As a result, fine-scale biologging studies often rely on energetic proxies such as dynamic body acceleration to estimate energetic output [54]. While these comparisons are valuable, without careful calibration, only relative energy expenditure conclusions can be made. Our low-cost custom-made swim tunnel respirometer offers a solution to collect energetics data for other larger species (electronic supplementary material). These data are a more direct connection between movement and metabolic output and provide insights into underlying physiology governing movement and habitat decisions.

Our presented metabolic model leveraged the scaling in mass-specific metabolic rates with body size and assumes much of the variation within the model stems from differences in locomotor costs at the varying swim intensities within the respirometer. Ideally, the model would incorporate swimming kinematic parameters to better account for movement costs across body sizes. To that end, efforts were made to construct a metabolic model as a function of swimming effort. First, there was a significant correlation in finning rate measured by the ITAG and mass-specific metabolic rate (electronic supplementary material, figure S1). However, this model does not account for jet propulsion, which is a key contributor to overall movement. We demonstrated that at high swimming intensities squid will decrease fin rates, so it is likely a finning-metabolic model alone would not accurately capture energy costs in the wild at high speeds when the jet becomes more prominent. Additionally, metabolic rate is often modelled as a function of swimming speed. However, the ITAG does not contain a speed sensor, which would limit the mapping of energy costs from laboratory to the field. Consequently, we isolated the data in which animals had similar behavioural state (i.e. metachronal fin-contributed locomotion) in laboratory and field and used respirometry to estimate physiological costs in the field. Our model may underestimate the metabolic cost of this fin-contributed behaviour since we were unable to test the full range of swimming speeds over which it is enacted. Future work should focus upon measuring energy costs across more diverse behaviours, including upwards swimming and high-amplitude jet propulsion gaits which have been shown to increase energy output [2].

Squid are posited to have a higher cost of transport and weaker swimming performance relative to niche-comparable fishes [16,30] (but see [19]). We show that squid may be compensating for these high-power requirements by selecting low-amplitude fin-contributed swimming for most of their time budgets (figure 4C). Fin-contributed swimming is more economical relative to jetting alone since the fins increase stability and enable forward thrust during mantle refilling [22,25]. High-amplitude jet movements, which have presumably higher energy cost, were only employed 4% of the time, indicating that slower, lower intensity movements were favoured. Mass-specific metabolic rates measured across laboratory swim trials were 292 ± 52 mg O_2_ kg^−1^ h^−1^, and these values are within the range of routine metabolic rates from marine fishes of similar mass (*Oncorhynchus nerka*: 486 ± 42 mg O_2_ kg^−1^ h^−1^, 2.6 kg, 12.5°C [55]; *Carcharhinus plumbeus*: 368 ± 66 mg O_2_ kg^−1^ h^−1^, 2 kg, 25°C [56]; *Carcharihinus limbatus*: 161 ± 19 mg O_2_ kg^−1^ h^−1^, 3.2 kg, 21.6°C; *Negaprion brevirostris*: 152 ± 30 mg O_2_ kg^−1^ h^−1^, 2.1−3.5 kg, 20.6°C [57]).

Squid may be further reducing energy output by swimming intermittently. Many marine and avian species that move in three dimensions exhibit intermittent movement strategies [58]. This swimming style is common for negatively buoyant species in which upwards active swimming (‘climbing’) is followed by an inactive decent (‘gliding’) phase. Extended gliding phases, where increased position in water column and potential energy are converted into horizontal movement, are energetically favourable compared to continuous swimming for many taxa [59–61]. *Loligo forbesii* employ climb-and-glide swimming during diel vertical migration and as a depth maintenance behaviour [25,35]. We found *L. forbesii* spent 18% of the time inactively gliding in the water column. Using the standard metabolic rate and hovering cost estimates (86–183 mg O_2_ kg^−1^ h^−1^) calculated from O’Dor *et al*. [50] on the same *L. forbesii* population, a 2 kg squid would reduce their mass-specific metabolic rate by over 48% by gliding, assuming no oxygen debts are being repaid (figure 5A,C). Data on the surrounding water currents and animal speed are needed to understand the ecological context of climb-and-glide swimming and to characterize its full energetic benefit. Interestingly, there were many descent gliding phases where fins remained active (figure 3B), and these sequences were classified as the fin-contributed swimming. This suggests descending periods are not always fin-inactive and vertical speed should be coupled with a behavioural metric to identify inactive gliding *in situ*.

Squid fin-contributed swimming and gliding required an estimated 3956 ± 767 mg O_2_ per day. Leveraging growth studies where squid (*Illex illecebrosus*) metabolic rates and feeding behaviours were systematically measured, we can translate movement costs to foraging needs to recoup energy output [62]. Using ecological conversion metrics (1 ml O_2_ to 4 mg fish), squid would need to consume an estimated 11.1 ± 1.4 g of fish or 0.97% of body weight every 24 hours to fuel these prevalent behaviours [25,62,63]. This estimate does not account for all energetic demands such as other high-amplitude movement behaviours, growth and developing gonads. However, it is conceivable that these squid exceed these energy requirements. Martins [39] conducted a stomach gut content analysis and found 53% of *L. forbesii* stomachs contained food, with most stomachs full to distended. Sampling was completed during daytime while *Loligo* are thought to primarily feed at night and full digestion occurs in *ca* 4 hours [64]. Indeed, squid may be far exceeding their foraging requirements to fuel their live-fast, die-young strategy where accelerated growth is heavily selected for at all ontogenic stages and heavy reproductive investment occurs after maturity [2,20,65]. The propensity for low-cost fin-contributed swimming and voracious foraging would support devoting a higher proportion of caloric intake to growth and could be key contributing factors to their apparent success in competing with marine fishes.

## 5. Conclusion

Squid are an abundant, ecologically keystone taxon found across diverse ocean biomes [7,14]. Despite this, most of our knowledge on their swimming mechanics and associated energetic costs stems from laboratory data. Here, we present systematic and repeatable workflows to characterize and quantify movement kinematics and energy costs of marine species. We also developed novel techniques to measure the movements of multiple body positions using a single biologging tag that can be extended to other species. To attribute specific energetic costs to measured movements in squid, we built a low-cost swim tunnel respirometer and developed bioenergetic models to map measured routine metabolic rates during metachronal fin-contributed swimming to energetic cost. The study taxon spent substantial time actively moving their fins, and by applying the calibrated model on movements naturally selected by animals *in situ*, we estimate that energetic cost of this movement pattern is similar to niche-comparable fishes. Higher intensity fin and jet movements are more energetically costly, but only accounted for significantly lower portions of the animal’s time budgets. While these high-acceleration movement patterns may be vital for key behaviours (not quantified here) such as foraging or avoiding predation, they come with presumed higher energetic costs and were correspondingly limited. These data suggest behavioural flexibility and diversity for mesopelagic squids and indicate that animals allocate higher proportions of their time budgets in energy-efficient locomotion. An important next step is to apply these kinematics, physiology and behaviour data on a seasonal time scale where squid are faced with different thermal regimes. Such data will provide vital predictive power of how a key prey species and energy link will contend with changing marine ecosystems.

## Data Availability

Data are available at https://erddap.bco-dmo.org/erddap/info/bcodmo_dataset_924340_v1/index.html [66]. Supplementary material is available online [67].

## References

[1] Robson AA, Chauvaud L, Wilson RP, Halsey LG. 2012 Small actions, big costs: the behavioural energetics of a commercially important invertebrate. J. R. Soc. Interface **9**, 1486–1498. (10.1098/rsif.2011.0713) 22219397 PMC3367807

[2] Webber DM, Aitken JP, O’Dor RK. 2000 Costs of locomotion and vertic dynamics of cephalopods and fish. Physiol. Biochem. Zool. **73**, 651–662. (10.1086/318100) 11121340

[3] Di Santo V. 2022 EcoPhysioMechanics: integrating energetics and biomechanics to understand fish locomotion under climate change. Integr. Comp. Biol. **62**, 711–720. (10.1093/icb/icac095) 35759407 PMC9494520

[4] Block BA *et al*. 2011 Tracking apex marine predator movements in a dynamic ocean. Nature **475**, 86–90. (10.1038/nature10082) 21697831

[5] Hussey NE *et al*. 2015 Aquatic animal telemetry: a panoramic window into the underwater world. Science **348**, 1255642. (10.1126/science.1255642) 26068859

[6] Chung H, Lee J, Lee WY. 2021 A review: marine bio-logging of animal behaviour and ocean environments. Ocean Sci. J. **56**, 117–131. (10.1007/s12601-021-00015-1)

[7] Clarke MR. 1996 Cephalopods as prey III. Phil. Trans. R. Soc. Lond. B **351**, 1053–1065. (10.1098/rstb.1996.0093)

[8] Hunsicker ME, Essington TE, Watson R, Sumaila UR. 2010 The contribution of cephalopods to global marine fisheries: can we have our squid and eat them too? Fish Fish. **11**, 421–438. (10.1111/j.1467-2979.2010.00369.x)

[9] Hoving HT *et al*. 2013 Extreme plasticity in life-history strategy allows a migratory predator (jumbo squid) to cope with a changing climate. Glob. Chang. Biol. **19**, 2089–2103. (10.1111/gcb.12198) 23505049

[10] Rosas-Luis R, Salinas-Zavala CA, Koch V, Luna PDM, Morales-Zárate MV. 2008 Importance of jumbo squid Dosidicus gigas (Orbigny, 1835) in the pelagic ecosystem of the central Gulf of California. Ecol. Model. **218**, 149–161. (10.1016/j.ecolmodel.2008.06.036)

[11] Seibel BA, Häfker NS, Trübenbach K, Zhang J, Tessier SN, Pörtner HO, Rosa R, Storey KB. 2014 Metabolic suppression during protracted exposure to hypoxia in the jumbo squid, Dosidicus gigas, living in an oxygen minimum zone. J. Exp. Biol. **217**, 2555–2568. (10.1242/jeb.100487) 24855676

[12] Doubleday ZA *et al*. 2016 Global proliferation of cephalopods. Curr. Biol. **26**, R406–R407. (10.1016/j.cub.2016.04.002) 27218844

[13] Roper CFE, Young RE. 1975 Vertical distribution of pelagic cephalopods. Smiths. Contrib. Zool. 1–51. (10.5479/si.00810282.209)

[14] Hanlon RT, Messenger JB. 2018 Cephalopod behaviour, 2nd edn. Cambridge, UK: Cambridge University Press.

[15] Pörtner HO, Zielinski S. 1998 Environmental constraints and the physiology of performance in squids. South Afr. J. Mar. Sci. **20**, 207–221. (10.2989/025776198784126421)

[16] O’Dor RK. 1982 Respiratory metabolism and swimming performance of the squid, Loligo opalescens. Can. J. Fish. Aquat. Sci. **39**, 580–587. (10.1139/f82-082)

[17] O’Dor RK, Webber DM. 1991 Invertebrate athletes: trade-offs between transport efficiency and power density in cephalopod evolution. J. Exp. Biol. **160**, 93–112. (10.1242/jeb.160.1.93)

[18] Webber DM, O’Dor RK. 1986 Monitoring the metabolic rate and activity of free-swimming squid with telemetered jet pressure. J. Exp. Biol. **126**, 205–224. (10.1242/jeb.126.1.205)

[19] Bartol IK, Mann R, Patterson MR. 2001 Aerobic respiratory costs of swimming in the negatively buoyant brief squid Lolliguncula brevis. J. Exp. Biol. **204**, 3639–3653. (10.1242/jeb.204.21.3639) 11719530

[20] O’Dor RK, Webber DM. 1986 The constraints on cephalopods: why squid aren’t fish. Can. J. Zool. **64**, 1591–1605. (10.1139/z86-241)

[21] Gladman NW, Askew GN. 2024 The contractile efficiency of the mantle muscle of European common cuttlefish (Sepia officinalis) during cyclical contractions. J. Exp. Biol. **227**, jeb249297. (10.1242/jeb.249297) 39297692 PMC11583979

[22] Bartol IK, Krueger PS, Jastrebsky RA, Williams S, Thompson JT. 2015 Volumetric flow imaging reveals the importance of vortex ring formation in squid swimming tail-first and arms-first. J. Exp. Biol. **219**, 392–403. (10.1242/jeb.129254) 26643088

[23] Gladman NW, Askew GN. 2023 The hydrodynamics of jet propulsion swimming in hatchling and juvenile European common cuttlefish, Sepia officinalis. J. Exp. Biol. **226**, jeb246225. (10.1242/jeb.246225) 37655637 PMC10560557

[24] Wells MJ, O’Dor RK. 1991 Jet propulsion and the evolution of the cephalopods. Bull. Mar. Sci **49**, 419–432.

[25] Hoar JA, Sim E, Webber DM, O’Dor RK. 1994 The role of fins in the competition between squid and fish. In Mechanics and physiology of animal swimming (eds L Maddock, Q Bone, J Rayner), pp. 27–43. Cambridge, UK: Cambridge University Press.

[26] Bartol IK, Ganley AM, Tumminelli AN, Krueger PS, Thompson JT. 2022 Vectored jets power arms-first and tail-first turns differently in brief squid with assistance from fins and keeled arms. J. Exp. Biol. **225**, b244151. (10.1242/jeb.244151) 35786780

[27] Bartol IK, Krueger PS, Stewart WJ, Thompson JT. 2009 Hydrodynamics of pulsed jetting in juvenile and adult brief squid Lolliguncula brevis: evidence of multiple jet 'modes' and their implications for propulsive efficiency. J. Exp. Biol. **212**, 1889–1903. (10.1242/jeb.027771) 19483007

[28] Wells MJ. 1990 Oxygen extraction and jet propulsion in cephalopods. Can. J. Zool. **68**, 815–824. (10.1139/z90-117)

[29] Stewart WJ, Bartol IK, Krueger PS. 2010 Hydrodynamic fin function of brief squid, Lolliguncula brevis. J. Exp. Biol. **213**, 2009–2024. (10.1242/jeb.039057) 20511514

[30] O’Dor RK. 1988 The forces acting on swimming squid. J. Exp. Biol. **137**, 421–442. (10.1242/jeb.137.1.421)

[31] O’Dor RK. 1992 Big squid in big currents. South Afr. J. Mar. Sci. **12**, 225–235.

[32] Mooney TA, Katija K, Shorter KA, Hurst T, Fontes J, Afonso P. 2015 ITAG: an eco-sensor for fine-scale behavioral measurements of soft-bodied marine invertebrates. Anim. Biotelemetry **3**, 14. (10.1186/s40317-015-0076-1)

[33] Duque Londono C, Cones SF, Deng J, Wu J, Yuk H, Guza DE, Mooney TA, Zhao X. 2024 Bioadhesive interface for marine sensors on diverse soft fragile species. Nat. Commun. **15**, 2958. (10.1038/s41467-024-46833-4) 38627374 PMC11021473

[34] Flaspohler GE, Caruso F, Mooney TA, Katija K, Fontes J, Afonso P, Shorter KA. 2019 Quantifying the swimming gaits of veined squid (Loligo forbesii) using bio-logging tags. J. Exp. Biol. **222** 1–13. (10.1242/jeb.198226) 31636155

[35] Cones S, Zhang D, Shorter K, Katija K, Mann D, Jensen F, Fontes J, Afonso P, Mooney T. 2022 Swimming behaviors during diel vertical migration in veined squid Loligo forbesii. Mar. Ecol. Prog. Ser. **691**, 83–96. (10.3354/meps14056)

[36] Gilly W *et al*. 2006 Vertical and horizontal migrations by the jumbo squid Dosidicus gigas revealed by electronic tagging. Mar. Ecol. Prog. Ser. **324**, 1–17. (10.3354/meps324001)

[37] Gilly WF, Zeidberg LD, Booth JAT, Stewart JS, Marshall G, Abernathy K, Bell LE. 2012 Locomotion and behavior of Humboldt squid, Dosidicus gigas, in relation to natural hypoxia in the Gulf of California, Mexico. J. Exp. Biol. **215**, 3175–3190. (10.1242/jeb.072538) 22915711

[38] Afonso P *et al*. 2020 The Azores: a mid-Atlantic hotspot for marine megafauna research and conservation. Front. Mar. Sci. **6**, 826. (10.3389/fmars.2019.00826)

[39] Martins HR. 1982 Biological studies of the exploited stock of Loligo forbesii (Mollusca: Cephalopoda) in the Azores. J. Mar. Biol. Assoc. UK **62**, 799–808.

[40] Porteiro FM, Martins HR. 1994 Biology of Loligo forbesii Steenstrup, 1856 (Mollusca: Cephalopoda) in the Azores: sample composition and maturation of squid caught by jigging. Fish. Res. **21**, 103–114. (10.1016/0165-7836(94)90098-1)

[41] Simões A, Duarte R, Alves M. 1997 A pilot ocean monitoring site at Azores islands. Elsevier Oceanogr. Ser. 444–451. (10.1016/s0422-9894(97)80054-2)

[42] Sigurd B, Hanlon RT. 1983 A review of the laboratory maintenance, rearing and culture of cephalopod molluscs. Mem. Natl Mus. Vic. **44**, 147–186. (10.24199/j.mmv.1983.44.11)

[43] Gonçalves JM, Porteiro FM, Cardigos F, Martins HR, Pham CK. 2009 The Azorean Loligo forbesii (Cephalopoda: Loliginidae) in captivity: transport, handling, maintenance, tagging and survival. Mar. Biodivers. Rec. **2**, e120. (10.1017/s1755267209001250)

[44] Cones SF, Jézéquel Y, Ferguson S, Aoki N, Mooney TA. 2022 Pile driving noise induces transient gait disruptions in the longfin squid (Doryteuthis pealeii). Front. Mar. Sci. **9**, 1070290. (10.3389/fmars.2022.1070290)

[45] MacDonald BA, Thompson RJ. 1986 Influence of temperature and food availability on the ecological energetics of the giant scallop Placopecten magellanicus. Mar. Biol. **93**, 37–48. (10.1007/BF00428653) 28312517

[46] Belman BW. 1978 Respiration and the effects of pressure on the mesopelagic vertically migrating squid Histioteuthis heteropsis. Limnol. Oceanogr. **23**, 735–739. (10.4319/lo.1978.23.4.0735)

[47] Seibel BA, Thuesen EV, Childress JJ, Gorodezky LA. 1997 Decline in pelagic cephalopod metabolism with habitat depth reflects differences in locomotory efficiency. Biol. Bull. **192**, 262–278. (10.2307/1542720) 28581868

[48] Di Santo V, Kenaley CP, Lauder GV. 2017 High postural costs and anaerobic metabolism during swimming support the hypothesis of a U-shaped metabolism-speed curve in fishes. Proc. Natl Acad. Sci. USA **114**, 13048–13053. (10.1073/pnas.1715141114) 29158392 PMC5724281

[49] Anderson E, Demont ME. 2005 The locomotory function of the fins in the squid Loligo pealei. Mar. Freshw. Behav. Physiol. **38**, 169–189. (10.1080/10236240500230765)

[50] O’Dor RK, Hoar JA, Webber DM, Carey FG, Tanaka S, Martins HR, Porteiro FM. 1995 Squid (Loligo forbesii) performance and metabolic rates in nature. Mar. Freshw. Behav. Physiol. **25**, 163–177. (10.1080/10236249409378915)

[51] Bartol IK, Ganley AM, Tumminelli AN, Bartol SM, Thompson JT, Krueger PS. 2023 Turning performance and wake dynamics of neritic squids. Mar. Biol. **170**, 73. (10.1007/s00227-023-04214-3)

[52] Li DH, Bartol IK, Gilly WF. 2023 Hydrodynamic diversity of jets mediated by giant and non-giant axon systems in brief squid. Integr. Comp. Biol. **63**, 1266–1276. (10.1093/icb/icad086) 37381578

[53] Ganley AM, Krueger PS, Bartol IK. 2023 Faster is not always better: turning performance trade-offs in the inshore squids Doryteuthis pealeii and Illex illecebrosus. J. Exp. Mar. Biol. Ecol. **565**, 151913. (10.1016/j.jembe.2023.151913)

[54] Gleiss AC, Wilson RP, Shepard ELC. 2011 Making overall dynamic body acceleration work: on the theory of acceleration as a proxy for energy expenditure. Methods Ecol. Evol. **2**, 23–33. (10.1111/j.2041-210x.2010.00057.x)

[55] Wagner GN, Kuchel LJ, Lotto A, Patterson DA, Shrimpton JM, Hinch SG, Farrell AP. Routine and active metabolic rates of migrating adult wild sockeye salmon (Oncorhynchus nerka Walbaum) in seawater and freshwater. Physiol. Biochem. Zool. **79**, 100–108. (10.1086/498186) 16380931

[56] Dowd WW, Brill RW, Bushnell PG, Musick JA. 2016 Standard and routine metabolic rates of juvenile sandbar sharks (Carcharhinus plumbeus), including the effects of body mass and acute temperature change. Fish. Bull. **104**, 323–331.

[57] Lear KO, Whitney NM, Brewster LR, Morris JJ, Hueter RE, Gleiss AC. 2017 Correlations of metabolic rate and body acceleration in three species of coastal sharks under contrasting temperature regimes. J. Exp. Biol. **220**, 397–407. (10.1242/jeb.146993) 27852751

[58] Gleiss AC *et al*. 2011 Convergent evolution in locomotory patterns of flying and swimming animals. Nat. Commun. **2**, 352. (10.1038/ncomms1350) 21673673

[59] Bideler JJ, Weihs D. 1982 Energetic advantages of burst-and-coast swimming of fish at high speeds. J. Exp. Biol. **97**, 169–178. (10.1242/jeb.97.1.169) 7086338

[60] Weihs D. 1973 Optimal fish cruising speed. Nature **245**, 48–50. (10.1038/245048a0)

[61] Zhang D, Wang Y, Gabaldon J, Lauderdale LK, Miller LJ, Barton K, Shorter KA. 2023 Dynamics and energetics of bottlenose dolphin (Tursiops truncatus) fluke-and-glide gait. J. Exp. Biol. **226**, b245237. (10.1242/jeb.245237) 37345501

[62] Hirtle RWM, DeMont ME, O’Dor RK. 1981 Feeding, growth, and metabolic rates in captive short-finned squid, Illex illecebrosus, in relation to the natural population. J. Shellfish Res. **1**, 187–192.

[63] Paloheimo JE, Dickie LM. 1966 Food and growth of fishes. III. Relations among food, body size, and growth efficiency. J. Fish. Board Can. **23**, 1209–1248.

[64] Bidder AM. 1966 Feeding and digestion in cephalopods. In Physiology of Mollusca (eds KM Wilbur, CM Yonge), pp. 97–124. London, UK: Academic Press. (10.1016/b978-1-4832-3242-3.50009-4)

[65] Smith JM, Pierce GJ, Zuur AF, Martins H, Clara Martins M, Porteiro F, Rocha F. 2011 Patterns of investment in reproductive and somatic tissues in the loliginid squid Loligo forbesii and Loligo vulgaris in Iberian and Azorean waters. Hydrobiologia **670**, 201–221. (10.1007/s10750-011-0666-8)

[66] Cones S, Mooney TA. 2024 Squid tag movement data and environmental sampling acquired in November 2021 and May 2023 near Faial and Pico Islands, Azores archipelago. Biological and Chemical Oceanography Data Management Office (BCO-DMO). (10.26008/1912/bco-dmo.924340)

[67] Cones S, Formel N, Fontes J, Afonso P, Shorter KA, Graça G *et al*. 2025 Supplementary material from: Prominent Fin-Contributed Swimming in Squid (Loligo forbesii) Supports Efficient Movement in Seamount Habitats. Figshare. (10.6084/m9.figshare.c.7911716) PMC12289209 40708662

